# Evaluation of the Therapeutic Effect of a Flavonoid Prescription against Rabbit Hemorrhagic Disease* In Vivo*

**DOI:** 10.1155/2019/5201790

**Published:** 2019-04-04

**Authors:** Hongxu Du, Shuaibing Zhang, Miao He, Ke Ming, Jinli Wang, Wenjuan Yuan, Mingyu Qiao, Yi Wu, Deyun Wang, Yuanliang Hu, Jiaguo Liu

**Affiliations:** Institute of Traditional Chinese Veterinary Medicine and MOE Joint International Research Laboratory of Animal Health and Food Safety, College of Veterinary Medicine, Nanjing Agricultural University, Nanjing 210095, China

## Abstract

Rabbit hemorrhagic disease (RHD) is an acute, high fatal contagious disease induced by rabbit hemorrhagic disease virus (RHDV) with acute severe hepatic injury and causes huge economic loss worldwide. In order to develop an effective and reliable drug to treat this disease in clinic, a prescription formulated with baicalin, linarin, icariin, and notoginsenoside R1 (BLIN) according to the theory of syndrome differentiation and treatment in traditional Chinese veterinary medicine was applied to investigate its curative effects against RHD* in vivo*. The preliminary study results showed that BLIN prescription exerted good curative effect on RHD therapy. To further validate the curative effect and to investigate the possible related curative mechanisms of this drug, the survival rates, the plasma biochemical indexes of hepatic function, the plasma evaluation indexes of oxidative injury, and the RHDV gene expression levels were detected and then the correlation among these indexes was also analyzed. These results showed that BLIN prescription could significantly increase the survival rate, reduce the hepatic injury severity, alleviate the oxidative injury, and decrease the RHDV gene expression level in rabbits infected with RHDV. All these results indicate that BLIN prescription possesses outstanding curative effect against RHD, and the curative mechanism may be related to its antioxidant and anti-RHDV activities. Therefore, this prescription can be expected to be exploited into a new candidate for RHD therapy in clinic.

## 1. Introduction

Rabbit hemorrhagic disease (RHD), also known as rabbit plague, is an acute, high fatal contagious disease caused by rabbit hemorrhagic disease virus (RHDV) [1]. This disease was first reported in China in 1984 [2]. To date, it has rapidly spread across rabbit populations throughout Asia [3], Europe [4], Australia [5], and America [6] and is causing severe economic losses in rabbit breeding industry worldwide [7]. Immunizing adult rabbits with attenuated RHDV vaccine is the main strategy to prevent this disease. However, a suitable cell culture system for large-scale production of vaccine antigens of RHDV is still unavailable [8]. Therefore, tissue-inactivated vaccine is most commonly used in clinic at present [9]. Although tissue-inactivated vaccine plays an important role in controlling RHD, this traditional vaccine also has a disadvantage of the easiness to spread the virus. What is worse, there are no effective treatment drugs available in clinic, once the clinical case emerged [10]. Hence, it is urgent to develop an effective drug to resist this disease.

Traditional Chinese medicine (TCM) has a long history of clinical application taking the form of prescription to treat various human and animal diseases in China and some other Asian countries [11, 12]. In recent years, a large number of studies have shown that TCM is an important strategy for viral diseases therapy. Ma* et al*. discovered San Wu Huangqin Decoction could inhibit influenza a/PR/8/34 (H1N1) virus infection* in vitro* and* in vivo* [13]. Research of Hsieh* et al.* demonstrated that GanLuSiaoDuYin could inhibit enterovirus 71 replication, translation, and cell apoptosis induced by virus [14]. With the development of the TCM chemistry, more and more bioactive ingredients are being isolated and identified. Therefore, in order to avoid the difference in clinical efficacy caused by differences in the quality of the TCM materials, an increasing number of researchers are trying to use TCM bioactive ingredients to formulate TCM prescriptions. Zhang* et al*. reported that a flavonoid prescription composed of epimedium flavones, sanchi flavones, wild dendranthema flower flavones, and Baikal skullcap root flavones could significantly inhibit Newcastle disease virus (NDV) infection and improve the protective rate of the Newcastle disease (ND) vaccine [15]. Furthermore, Fan* et al*. also discovered that epimedium polysaccharide-propolis flavone prescription could effectively resist the immunosuppression induced by cyclophosphamide on chickens [16].

According to the theory of syndrome differentiation and treatment in traditional Chinese veterinary medicine, RHD is considered to be the syndrome of blood-heat and liver-wind agitations [17]. Baicalin-linarin-icariin-notoginsenoside R1 (BLIN) prescription is a TCM ingredients prescription, which is formulated to clear liver-heat, extinguish liver-wind, cool blood, and eliminate the evil according to the theory of traditional Chinese veterinary medicine [18]. In particular, our previous research has demonstrated that BLIN prescription could exert significant curative effects on duck viral hepatitis (DVH), which is a disease of the same syndrome as RHD, and showed the similar clinical manifestations, such as screaming, loss of appetite, convulsion, and hepatic failure. However, the efficacy of this prescription on RHD is unknown. Therefore, we conducted this research to investigate the curative effect and the possible relevant mechanisms of this prescription on RHD therapy.

## 2. Materials and Methods

### 2.1. Ethics Statement

Specific-pathogen-free two-month-old New Zealand rabbits were supplied by Jiangsu Academy of Agricultural Science. The average weight of the rabbits used in the experiments was 2.55 ± 0.21 kg. All animal experiments conformed to the Guide for the Care and Use of Laboratory Animals published by the US National Institutes of Health (NIH Publication, Eighth edition, 2011) and were approved by the Nanjing Agricultural University Animal Care Committee (No. 20130093, 2013). Special care was taken to minimize number and suffering of the animals. The rabbits were housed in individual cages and received humane care according to the Principles of Laboratory Animal Care. The laboratory temperature was maintained at 24.0 ± 1.0°С. During the entire experiment, rabbits were carefully nursed to reduce all types of stress, and each process was carried out strictly in accordance with the regulations of the animal protection committee. To ameliorate suffering, rabbits with typical clinical symptoms (such as screaming, struggling, convulsion, and running around) who were not expected to survive were humanely euthanized. All steps complied with AVMA Guidelines for the Euthanasia of Animals (2013 Edition).

### 2.2. Drugs and Virus

The preparation process of BLIN prescription was the same as our previous study [18]. In short, BLIN prescription was prepared based on four flavonoids: baicalin (98 %), linarin (95%), icariin (98%), and notoginsenoside R1 (93%). The proportion of them in the prescription was 4:2:16:1, respectively, according to the theory of monarch, minister, and assistant guide [19, 20]. For experiments* in vivo*, the prescription was diluted into 20 mg (net content of the drugs)/mL with 1% added glycerinum distilled water, and the PH was adjusted with 5.6% NaHCO_3_ to 7.5.

RHDV (FJ794180 strain) solution (HA = 2^8^) for challenge experiments was supplied by Jiangsu Academy of Agricultural Science.

### 2.3. Reagents

Superoxide dismutase (SOD) assay kit (Lot no. 20141210), Glutathione peroxidase (GSH-Px) assay kit (Lot no. 20150107), Catalase (CAT) assay kit (Lot no. 20141216), Malondialdehyde (MDA) assay kit (Lot no. 20150107), and total antioxidant capacity (T-AOC) assay kit (Lot no. 20150115) were purchased from Nanjing Jiancheng Bioengineering Institute. RNAiso Plus Reagent (Lot no. 9108), PrimeScript™ RT Master Mix Kit (Lot no. AK 3101), and SYBR® Premix Ex Taq™ Kit (Tli RNaseH Plus) (Lot no. AK 5303) were the products of Takara Biotechnology Co., Ltd.

### 2.4. Preliminary Study on the Therapeutic Effect of BLIN Prescription against RHD

Forty-five rabbits were randomly divided into three groups: BLIN group, virus control (VC) group, and blank control (BC) group (separately reared). After the animals adapted to the environment, the rabbits of the BLIN group were treated with BLIN solution by drinking water at the dosage of 15 mg/kg body weight once a day for 3 days. The rabbits in the BC and VC groups were treated with the same dose of solvent-added solution. On the third day, each rabbit of the BLIN and VC groups was subcutaneously injected with 0.2 mL of RHDV solution after the drug administration. Thereafter, rabbits in the BLIN group continued to be treated with BLIN prescription for another 3 days. During the experiment, once a rabbit died, it would be dissected and the pathological changes would be record immediately. Only when the pathological changes were identified as RHD was the death counted. To evaluate the clinical therapeutic effect of the prescription, the number of the final survival rabbits in each group was recorded. The survival rate in each group was calculated according to the following formula: survival rate (%) = the number of surviving rabbits/the number in the sample group × 100%.

### 2.5. Validation Study on the Therapeutic Effect of BLIN Prescription against RHD

According to the feeding and management procedures described above, forty-five rabbits were randomly divided into three groups: BLIN group, VC group, and BC group (separately reared). The following challenge and treatment procedures are the same as above. In order to monitor the indexes of hepatic function and oxidative injury, blood samples were randomly taken from four rabbits per group at the initial phase (8 hpi and 24 hpi) (hpi: hours post-infection) and recovery phase (96 hpi). During the experiment, the health status of the rabbits was monitored 2 times per day and the number of deaths in each group was recorded at the same time. Once a rabbit died, it would be dissected and the pathological changes would be recorded immediately. Only when the pathological changes were identified as RHD was the death counted. Dead rabbits were executed and disposed of in bio-safety containers in accordance with local standard protocols. Ultimately, the survival data collected above in each group were used for survival curve analysis.

### 2.6. Detection of the Changes of the Plasma Hepatic Function Indexes

The plasma levels of alanine transaminase (ALT), aspartate transaminase (AST), alkaline phosphatase (ALP), and lactate dehydrogenase (LDH) were tested by automatic biochemistry analyzer (7180 Automatic Analyzer, HITACHI, Japan).

### 2.7. Evaluation of the Changes of the Plasma Antioxidase Activities and the Total Antioxidant Capacity

The plasma antioxidase (SOD, GSH-Px, and CAT) activities and the total antioxidant capacity of each group at different time point were tested by corresponding assay kit. All the test operations were strictly in accordance with the instructions.

### 2.8. Evaluation of the Changes of the Plasma MDA Contents

The plasma MDA contents of each group at different time points were detected by the MDA assay kit. All the test operations were strictly in accordance with the instructions.

### 2.9. Relative RHDV Gene Expression Levels in Each Group

Total RNA was extracted from whole blood samples with RNAiso Plus Reagent according to introduction of the kit. Then cDNA was synthesized by PCR instrument (2720 Thermal Cycler PCR instrument, Applied Biosystems, USA) with Prime Script™ RT Master Mix Kit. The cycling program was 37°С for 15 min, 85°С for 5 s, and 4°С for 7 min. Ultimately, semiquantitative analysis of the virus gene expression level was conducted with RT-PCR instrument (StepOnePlus™ Real Time PCR instrument, Applied Biosystems, USA) by using SYBR® Premix Ex Taq™ Kit (Tli RNaseH Plus). The primers of RHDV for the RT-PCR reaction were designed based on RHDV capsid protein VP60 sequences (GenBank No: AY269825) and primers of *β*-actin were designed based on* Oryctolagus cuniculus* actin (NM-001101683). The primer sequences were as follows: RHDV forward: 5'-CCATCATGTTCGCGTCTGTT-3'; RHDV reverse: 5'-GGGCGTACGTCAATGAGTTC-3'; *β*-actin forward: 5'-TGGCATCCTGACGCTCAA-3'; *β*-actin reverse: 5'-TCGTCCCAGTTGGTCACGAT-3'.

### 2.10. Correlation Analysis

The correlation analyses among the survival rate, hepatic function indexes, oxidative damage evaluation indexes, and relative RHDV gene expression level at 96 hpi were performed by Pearson's correlation coefficient using SPSS Software Package v.20.0.

### 2.11. Statistical Analysis

Duncan's multiple range test was used to analyze the difference among groups with the software SPSS v.20.0. Kaplan-Meier survival curves were generated in GraphPad Prism 6 (GraphPad Software Inc.) for statistical analysis of the survival rates. The 2^−ΔΔCT^ method [21] was used to analyze the relative RHDV gene expression data. Results were expressed as means ± S.E. Significant differences were considered as* p* < 0.05.

## 3. Results

### 3.1. Results of the Preliminary Study on the Therapeutic Effect of BLIN Prescription

Table 1 presents the results of the survival rates in each group in preliminary study. As the rabbits of the BC group were not challenged with RHDV and fed separately, no rabbits died throughout the experiment. As for the VC group, all the rabbits died with the typical pathological changes of RHD, so the survival rate is 0, which is significantly lower than that of the BC group (*p* < 0.05). In contrast, the survival rate of the BLIN group was increased up to 53.3% compared with that of the VC group and the difference between these two groups was statistically significant (*p* < 0.05).

### 3.2. Results of Validation Study on the Therapeutic Effect of BLIN Prescription

**Figure 1:**
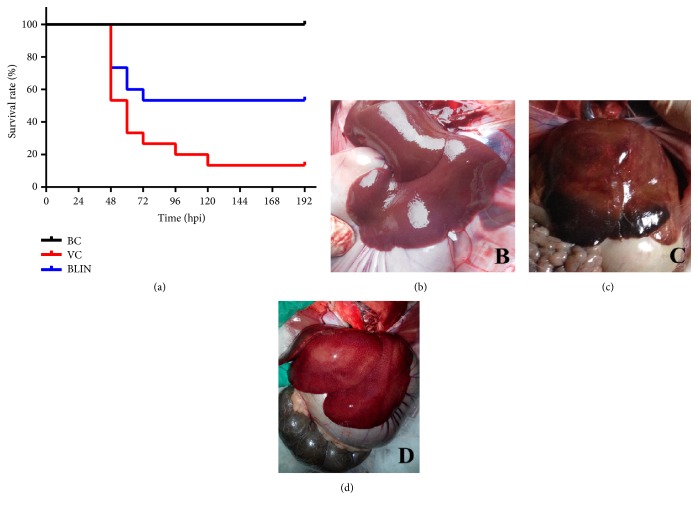
The survival curve (a) and the visual hepatic pathological changes of the BC group (b), the VC group (c), and the BLIN group (d).

Figure 1(a) illustrates the survival curves of each group during the validation study. As the rabbits of the BC group were not challenged with RHDV and fed separately, no rabbits died throughout the experiment. After the rabbits were challenged for 32 h, no dead cases were found in all the three groups. However, more than half of the rabbits in the VC group died at 48 hpi. Ultimately, the number of the surviving rabbits and the survival rate of the VC group are 2 and 13.3%, respectively, which are significantly lower than those of the BC group (*p* < 0.05). Similar to the results of the preliminary study shown above, the number of surviving rabbits and survival rate were significantly increased to 8 and 53.3%, respectively (*p* < 0.05), when the RHDV infected rabbits were treated with BLIN prescription.

### 3.3. Results of the Visual Hepatic Pathological Changes

The visual hepatic pathological changes of the BC, VC, and BLIN groups are illustrated in Figures 1(b), 1(c), and 1(d). As shown in Figure 1(b), the liver surface was lubricious and no pathological changes were observed in the BC group. However, for the rabbit liver of the VC group (Figure 1(c)), hepatic congestion and hepatomegaly appeared, the color of the liver turned dark red or purple, and the texture of the liver became brittle. On the contrary, the visual pathological changes were obviously alleviated and small congestion was found on the liver surface, when the rabbits infected with RHDV were treated with BLIN prescription (Figure 1(d)). Moreover, the color of rabbit liver of the BLIN group was much closer to that of the BC group compared with that of the VC group.

### 3.4. Results of the Hepatic Function Test

**Figure 2:**
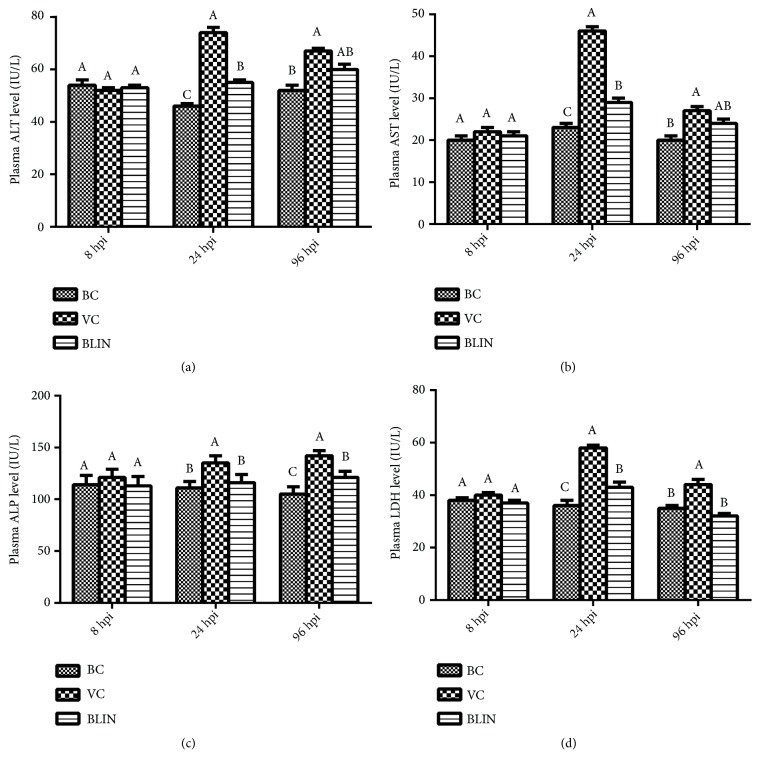
*The levels of the plasma hepatic function indexes of each group at 8 hpi, 24 hpi, and 96 hpi*. (A-C) Bars in the same index at the same time point without the same superscripts differ significantly (*p *<0.05).

Figure 2 shows the results of the hepatic function indexes of the BC, VC, and BLIN groups at 8 hpi, 24 hpi, and 96 hpi. At 8 hpi, the plasma ALT, AST, ALP, and LDH levels of each group were at the same level and showed no significant difference among them (*p *> 0.05). At 24 hpi, all the hepatic function indexes of the VC group were increased and were significantly higher than those of the BC group (*p *< 0.05). Meanwhile, compared with the VC group, all those indexes were significantly decreased in the BLIN group (*p *< 0.05). However, the plasma ALT, AST, and LDH levels of the BLIN group were still significantly higher than those of the BC group at this time point (*p *< 0.05). Although the ALT, AST, and LDH levels of the VC group were obviously reduced at 96 hpi, those indexes of the VC group were still significantly higher than those of the BC group (*p *< 0.05). As for the indexes of ALT and AST, there was no significant difference between the VC and BLIN groups (*p *> 0.05), but the ALT and AST levels of BLIN group were much lower than those of the VC group. As for the indexes of the ALP and LDH, those two indexes of the BLIN group were significantly decreased compared to those of the VC group (*p *< 0.05).

### 3.5. Results of the Changes of the Plasma Antioxidase Activities and the Total Antioxidant Capacity

**Figure 3:**
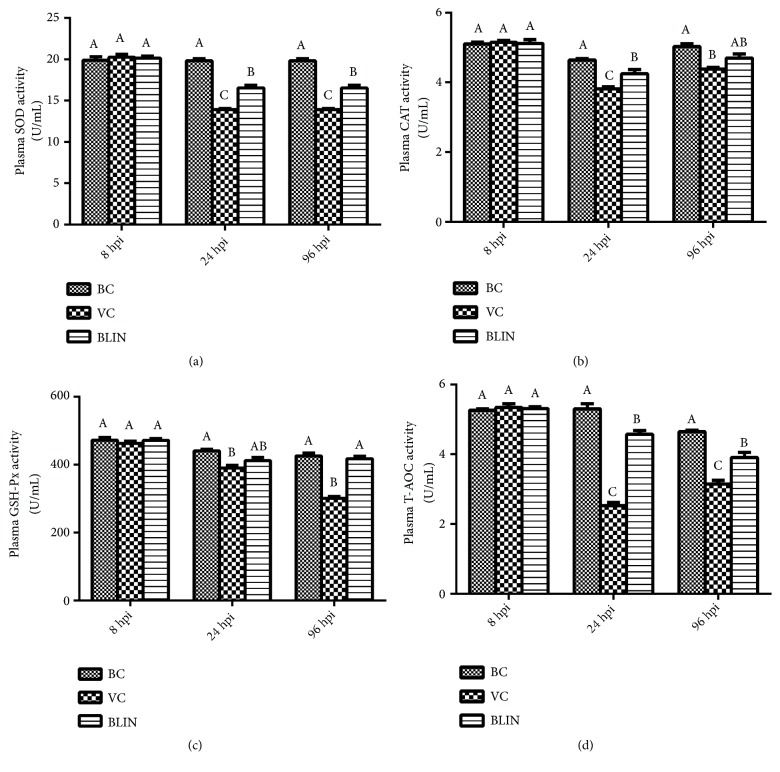
*The activities of plasma antioxidase and the total antioxidant capacity of each group at 8 hpi, 24 hpi, and 96 hpi*. (A-C) Bars in the same index at the same time point without the same superscripts differ significantly (*p *<0.05).

Figure 3 shows the results of plasma SOD, CAT, GSH-Px, and T-AOC activities in each group at 8 hpi, 24 hpi, and 96 hpi. At 8 hpi, the plasma SOD, CAT, GSH-Px, and T-AOC activities of the VC group were not distinct from those of the BC and BLIN groups. At 24 hpi, the activities of SOD, CAT, and T-AOC of the VC and BLIN groups were significantly lower than those of the BC group (*p* < 0.05), and their activities in the VC group were the lowest among the three groups and were significantly lower than those of the BLIN group (*p *< 0.05). At 96 hpi, the plasma SOD, GSH-Px, and T-AOC activities of the BLIN group were reinstated close to the normal level but were still significantly higher than those of the VC group (*p *< 0.05). Meanwhile, the activity of CAT in the BC group was significantly higher than that of the VC group (*p *< 0.05), but no significant difference existed between the BC group and the BLIN group (*p *> 0.05) and between the BLIN group and the VC group (*p *> 0.05).

### 3.6. Results of the Changes of the Plasma MDA Contents

**Figure 4:**
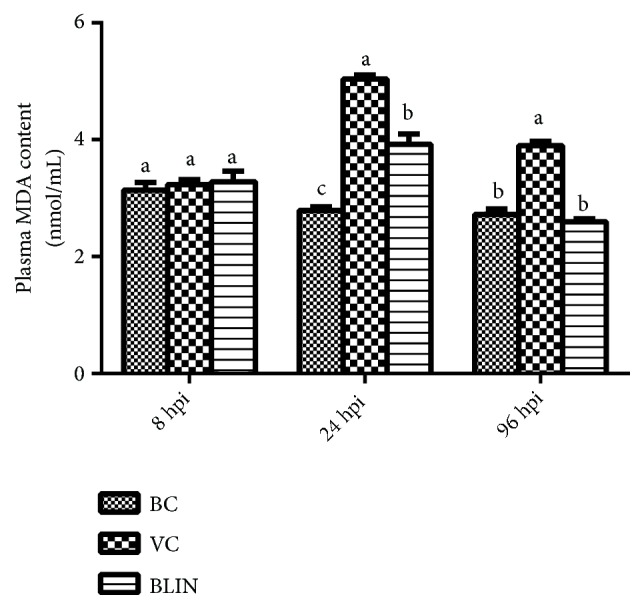
*The plasma MDA contents of each group at 8 hpi, 24 hpi, and 96 hpi*. (a-c) Bars in the same index at the same time point without the same superscripts differ significantly (*p *<0.05).

The plasma MDA contents in each group at 8 hpi, 24 hpi, and 96 hpi are illustrated in Figure 4. At 8 hpi, the plasma MDA contents of the BC, VC, and BLIN groups showed no significant difference (*p *> 0.05). At 24 hpi, the plasma MDA contents of the BLIN and VC groups increased a lot and were significantly higher than that of the BC group (*p *< 0.05). Meanwhile, the plasma MDA content of the VC group was significantly higher than that of the BLIN group (*p *< 0.05). At 96 hpi, the plasma MDA content of the BLIN group returned to normal level, while the content of this index of the VC group was still significantly higher than that of the other two groups (*p *< 0.05).

### 3.7. Results of Relative Expression of RHDV Gene in Blood

**Figure 5:**
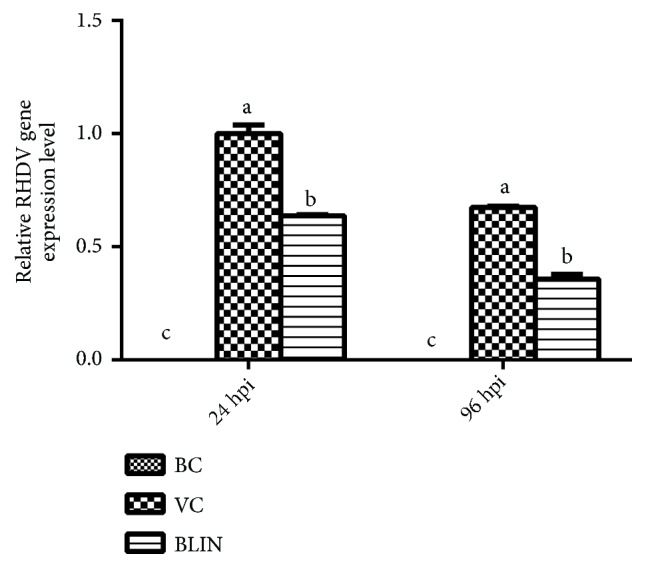
*Relative RHDV gene expression level of each group at 24 hpi and 96 hpi*. (a-c) Bars in the same index at the same time point without the same superscripts differ significantly (*p *<0.05).

The relative RHDV gene expression levels in each group at 24 hpi and 96 hpi are illustrated in Figure 5. RHDV gene expression level of the VC group at the 24 hpi was set to 1. The RHDV gene was not detected in the BC group at the sampling time point, so the RHDV gene expression level of the BC group was 0. On the other hand, the RHDV gene expression level of the VC and BLIN groups was significantly higher than that of the BC group at all those sampling time points (*p* < 0.05). Moreover, at both 24 hpi and 96 hpi, the relative RHDV gene expression levels of the BLIN group were always significantly lower than that of the VC group (*p *< 0.05).

### 3.8. Results of the Correlation Analysis

The Pearson correlation coefficients among the survival rate, hepatic function indexes, relative RHDV gene expression level, and oxidative injury indexes at 96 hpi are listed in Table 2. As shown in the list, SOD, CAT, GSH-Px, and T-AOC were negatively correlated with ALT, AST, ALP, and LDH but were positively correlated with survival rate. On the contrary, MDA was positively correlated with ALT, AST, ALP, and LDH but was negatively correlated with survival rate. Meanwhile, RHDV gene expression level was significantly and positively correlated with ALT, AST, ALP, and LDH (*p* < 0.05) but was significantly and negatively correlated with survival rate (*p* < 0.05). In addition, ALT, AST, and ALP were also significantly and negatively correlated with survival rate (*p *< 0.05).

## 4. Discussion

Screening antiviral drugs through cell tests is a time-saving, inexpensive, effective, and reliable method. However, no suitable tissue culture system is available for RHDV proliferation [8]. Therefore, it greatly restricts the discovery process of the drugs against this disease* in vitro* and we can only conduct our experiments* in vivo*.

For fatal diseases, survival rate of animals after drug treatment is a key indicator for drug efficacy evaluation [22]. In order to investigate whether BLIN prescription could exert curative effect on RHD therapy, we performed the preliminary experiment* in vivo*. As the results show in Table 1, the final survival rate of the VC group was 0. However, the survival rate of the infected rabbits with BLIN prescription treatment increased up to 53.3%, which is significantly higher than that of the VC group (*p* < 0.05). These preliminary results demonstrate that BLIN prescription is a promising candidate for RHD therapy in clinic.

To further validate the therapeutic effect of the prescription and investigate the possible related curative mechanisms, another experiment was conducted* in vivo*. Similarly, the following survival rate results of the validation study also show that BLIN prescription can significantly decrease the high mortality rate induced by RHDV (Figure 1(a)). The consistency between these results further validates the effectiveness of the BLIN prescription on RHD treatment.

As one of the major histological changes, fulminant hepatic failure contributes greatly to the death of the rabbits infected with RHDV [23, 24]. Hence, it makes sense to improve the hepatic function in the treatment of this disease. ALT, AST, ALP, and LDH are important indexes for the assessment of hepatic injury [25, 26]. ALT mainly exists in the cytoplasm of the hepatocytes, and it will be released once the hepatocyte membranes are broken. Therefore, the plasma ALT level is the primary diagnostic index of hepatic injury [27]. AST is widely distributed in many kinds of organs. In general, the content of AST in the hepatocytes is the second largest. Since AST mainly exists in the mitochondria of the hepatocytes, plasma AST content will significantly increase only when hepatocytes are severely damaged. On the other hand, ALP and LDH are reported as the important indicators of hepatic injury [28]. In our experiment, the plasma AST, ALT, ALP, and LDH contents among the three groups were at the same level at 8 hpi (Figure 2). However, at 24 hpi (Figure 2), all the plasma hepatic function indexes that were tested in the VC group were significantly elevated compared with those of the BC group (*p* < 0.05). These results indicate that the hepatic injury appeared at 8 to 24 hours following RHDV infection. Meanwhile, the decrease of the ALT, AST, and LDH contents of the VC group at 96 hpi may suggest that the body is working to repair the hepatic injury at the recovery phase. However, all the hepatic function indexes of the VC group were still significantly lower than those of the BC group at 96 hpi (*p* < 0.05). These results may imply that the hepatic injury was still very serious, and the prognosis of the disease was poor in the VC group. In contrast, the decrease of these four indexes in the BLIN group at 24 hpi and 96 hpi may suggest that BLIN protected the liver from injury caused by RHDV infection. Meanwhile, the correlation analysis results also show that the hepatic function indexes (ALT, AST, ALP, and LDH) were negatively correlated with the survival rate (Table 2). These results suggest that the survival rate was negatively correlated with the hepatic injury, and the worse the injury, the lower the survival rate. Therefore, it is obvious that the curative effect of BLIN on RHD therapy was attributed to its hepatoprotective effect. However, what are the mechanisms of its hepatoprotective effect?

Many of evidences accumulated over the past decades suggested that patients with RNA virus infection are under oxidative injury [29]. RHDV infection is not an exception, and some recent researches also demonstrated that oxidative injury was an important pathological mechanism of RHDV-infected rabbits with serious hepatic injury [30, 31]. Oxidative injury refers to the damage caused by the imbalance of the free radical metabolism [32]. Under normal physiological conditions, free radicals are generated and eliminated continuously to keep the dynamic balance of the redox status [33]. However, once free radical production overwhelms antioxidant defense, it will result in oxidative stress and cause injury of the biological macromolecules and cells or even cell death [34]. As to antioxidant defense system, it includes two groups: one is the enzymatic antioxidant group and another one is the non-enzymatic antioxidant group, and the former plays the pivotal role. The enzymatic antioxidant group was mainly consisted of SOD, CAT, GSH-Px, and some other antioxidant enzymes [35]. For SOD, it can degrade superoxide radicals into hydrogen peroxide [36]. Then CAT and GSH-Px degrade hydrogen peroxide into H_2_O [37]. Their synergistic effects keep the balance of the redox status and protect cells and biological macromolecules from oxidative damage. In addition, for a more comprehensive evaluation of the total free radical scavenging capacity, the index of T-AOC (total antioxidant capacity) was also tested in our research. On the other hand, as the main product of lipid peroxidation, MDA is of great significance in reflecting the degree of lipid peroxidation injury and oxidative stress [38].

As the results illustrated in Figures 3 and 4, the plasma indexes of SOD, CAT, GSH-Px, T-AOC, and MDA among the three groups showed no significant differences at 8 hpi (*p *> 0.05). Apparently, the balance of the redox status had not been destroyed at this time point. When rabbits were challenged with RHDV for 24 h, the plasma SOD, CAT, GSH-Px, and T-AOC levels of the VC group were significantly decreased (*p* < 0.05), and the plasma MDA content of the VC group was significantly increased (*p* < 0.05). These results may indicate that the excessive free radicals overwhelmed the antioxidant defense and caused serious oxidative injury in the body. Although the levels of CAT, T-AOC, and MDA of the VC group were changed at 96 hpi compared with those indexes of the VC group at 24 hpi, the plasma SOD, CAT, GSH-Px, and T-AOC levels of the VC group remained the lowest among the three groups, and the plasma MDA content of the VC group was still the highest. These results may imply that the imbalance of the redox status had not been changed, as the disease progressed. Meanwhile, the correlation analysis results (Table 2) showed that the indexes of SOD, CAT, GSH-Px, and T-AOC were negatively correlated with the hepatic function indexes (ALT, AST, ALP, and LDH) and were positively correlated with the survival rate. The MDA content was positively correlated with the hepatic function indexes (ALT, AST, ALP, and LDH) and was negatively correlated with the survival rate. These results indicate that the hepatic injury and the mortality rate of this disease were positively correlated with the oxidative stress. That is to say, the higher the activities of the SOD, CAT, GSH-Px, and T-AOC and the lower the MDA content, the lower the hepatic injury and the mortality rate. Therefore, it is clear that oxidative injury made a great contribution to the hepatic injury induced by RHDV. Moreover, it is not difficult to understand the highest levels of the ALT, AST, ALP, and LDH and the mortality rate of the VC group among the three groups. On the contrary, for the BLIN group, the levels of plasma SOD, CAT, GSH-Px, and T-AOC were much higher than those of the VC group, and the plasma MDA content was significantly lower than that of the VC group (*p *< 0.05) at 24 hpi and 96 hpi. Moreover, all these oxidative injury evaluation indexes of the BLIN group were adjusted back to near-normal levels at 96 hpi. These results may suggest that BLIN prescription exerted good antioxidant activity against the oxidative injury of the rabbits infected with RHDV. Therefore, it is clear that one of the curative mechanisms of BLIN on RHD is based on its antioxidant activity.

As for viral diseases, the health status is closely related with the virus level in the body, and a higher virus level indicates a worse health status [39]. In this study, the dynamic RHDV gene expression levels in the blood were tested to reflect the virus level in the body. With the proliferation of the RHDV in the body, a certain number of RHDV pathogens were generated. So the RHDV gene was detected and reached a relatively high level at 24 hpi (Figure 5). However, for rabbits in the BLIN group, the relative RHDV gene expression level was significantly lower than that of the VC group at the same time point (*p* < 0.05). Although the relative RHDV gene expression levels of the VC and BLIN groups were decreased at 96 hpi, the RHDV gene could also be detected in both groups. The decrease of the RHDV gene expression level in the VC group at 96 hpi may suggest that RHDV was going through the natural process of being gradually cleaned out. Similar to 24 hpi, the RHDV gene expression level of the VC group remained the highest among the three groups and significantly higher than that of the BLIN group at 96 hpi (*p *< 0.05). All these results imply that the BLIN prescription could exert good anti-RHDV activity. Meanwhile, the correlation analysis results showed that relative RHDV gene expression level was positively correlated with the hepatic injury indexes and was negatively correlated with the survival rate (Table 2). It indicates that when the RHDV gene expression level in the blood increases, more hepatocytes are infected, leading to the exacerbation of the hepatic injury and the elevation of the mortality rate. Therefore, it is possible that BLIN could also exert its curative effect by inhibiting the proliferation of RHDV.

## 5. Conclusions

In a word, BLIN prescription was confirmed with superior curative effect on RHD* in vivo*. Research into the mechanism suggests that BLIN confers a curative effect that is closely linked to its antioxidant and anti-RHDV activities. All these results indicate that the BLIN prescription can be expected to be developed into a new candidate for RHD therapy.

## Figures and Tables

**Table 1:** The curative effect of the BLIN prescription in the preliminary study.

Group	Sample number	Number of survivors	Survival rate (%)
BC	15	15	100.0^a^
VC	15	0	0.0^c^
BLIN	15	8	53.3^b^

^a-c^Data within a column without the same superscripts differ significantly (*p* < 0.05). hpi: hours post-infection.

**Table 2:** The Pearson correlation coefficients among oxidative injury evaluation indexes, hepatic function indexes, survival rate, and RHDV gene expression level at 96 hpi.

	SOD	CAT	GSH-Px	TAOC	MDA	ALT	AST	ALP	LDH	Survival rate	RHDV gene expression level
SOD	1.000^*∗*^	0.998^*∗*^	0.863^*∗*^	0.997^*∗*^	-0.780	-0.986^*∗*^	-1.000^*∗*^	-0.990^*∗*^	-0.673	0.987^*∗*^	-0.999^*∗*^
CAT		1.000^*∗*^	0.890^*∗*^	1.000^*∗*^	-0.814	-1.000^*∗*^	-0.997^*∗*^	-0.996^*∗*^	-0.713	0.977^*∗*^	-1.000^*∗*^
GSH-Px			1.000^*∗*^	0.897^*∗*^	-0.989^*∗*^	-0.877^*∗*^	-0.855^*∗*^	-0.926^*∗*^	-0.955	0.771	-0.879^*∗*^
TAOC				1.000^*∗*^	-0.824	-0.999^*∗*^	-0.996^*∗*^	-0.997^*∗*^	-0.725	0.973^*∗*^	-0.999^*∗*^
MDA					1.000^*∗*^	0.797	0.770	0.862^*∗*^	0.988^*∗*^	-0.670	0.800
ALT						1.000^*∗*^	0.999^*∗*^	0.993^*∗*^	0.693	-0.982^*∗*^	1.000^*∗*^
AST							1.000^*∗*^	0.987^*∗*^	0.661	-0.990^*∗*^	0.999^*∗*^
ALP								1.000^*∗*^	0.772	-0.954^*∗*^	0.994^*∗*^
LDH									1.000^*∗*^	-0.546	0.696
Survival rate										1.000^*∗*^	-0.981^*∗*^
RHDV gene expression level											1.000^*∗*^

*∗* indicates a significant correlation (*p* < 0.05). hpi: hours post-infection.

## Data Availability

The data used to support the findings of this study are available from the corresponding author upon request.
